# Arthroplasty versus internal fixation for displaced femoral neck fractures in older patients with dementia: a target trial emulation study

**DOI:** 10.2340/17453674.2026.46936

**Published:** 2026-10-26

**Authors:** Simon Storgaard JENSEN, Per Hviid GUNDTOFT, Rob G H H NELISSEN, Jan-Erik GJERTSEN, Alma B PEDERSEN

**Affiliations:** 1 Department of Clinical Epidemiology, Aarhus University Hospital, Aarhus, Denmark; 2 Department of Clinical Medicine, Aarhus University, Aarhus, Denmark; 3 Department of Orthopaedic Surgery, Traumatology, Aarhus University Hospital, Aarhus, Denmark; 4 Department of Orthopaedics, Leiden Universitair Medisch Centrum, Leiden, The Netherlands; 5 Department of Orthopaedic Surgery, Haukeland University Hospital, Bergen, Norway; 6 Department of Clinical Medicine, University of Bergen, Bergen, Norway

## Abstract

**Background and purpose:**

Target trial emulation can improve design transparency in observational comparative-effectiveness studies. We applied the framework to compare the outcome after arthroplasty with that of internal fixation (IF) in patients with dementia and displaced femoral neck fracture regarding recorded reoperation, mortality, length of stay, and postoperative mobility.

**Methods:**

Using nationwide Danish registries, we emulated a target trial among patients aged ≥ 70 years. Patients were classified by index treatment (arthroplasty or IF) and analyzed according to the intention-to-treat estimand. Recorded reoperation in the Danish National Patient Registry within 365 days was the primary outcome. Secondary outcomes included major reoperation, mortality, length of stay, early mobilization, and mobility decline. Stabilized inverse probability of treatment weighting balanced measured covariates. We estimated weighted cumulative incidences accounting for competing death and weighted cause-specific hazard ratios (HRs) with 95% confidence intervals (CIs).

**Results:**

The emulated target trial included 3,161 patients, of whom 2,231 received arthroplasty and 930 IF. At 365 days, recorded reoperation occurred in 298/2,231 vs 68/930 patients receiving arthroplasty or IF, respectively; weighted incidences were 13% (CI 13–14) vs 7.6% (CI 7.0–8.3) (HR 0.52, CI 0.39–0.69). Major reoperation occurred in 191/2,231 vs 68/930; weighted incidences were 8.6% (CI 8.2–8.9) vs 7.6% (CI 7.1–8.2) (HR 0.84, CI 0.63–1.10). Death occurred in 1,040/2,231 vs 421/930; weighted estimates were 47% (CI 46–48) vs 43% (CI 42–44) (HR 0.86, CI 0.77–0.98). Length of stay was modestly longer after arthroplasty, whereas early mobilization and postoperative mobility were similar.

**Conclusion:**

IF was associated with fewer recorded reoperations and lower mortality than arthroplasty in routine care. Active-comparator target trial emulation enabled a transparent comparison of the treatment strategies, but residual confounding and differential outcome ascertainment limited causal and clinical interpretation.

Displaced femoral neck fractures (FNFs) are usually treated with cemented hemiarthroplasty or total hip arthroplasty because arthroplasty is associated with lower risks of postoperative complications and reoperation than internal fixation (IF) [1,2]. Nevertheless, Danish registry data indicates that a substantial proportion of patients continue to receive IF and that uncemented stems remain common among those treated with arthroplasty [1,3].

In routine care, treatment choice and deviation from guideline recommendations may reflect frailty, operative and anesthetic risk, local organizational circumstances, goals of care, and cognitive status [4-6]. Dementia affects approximately 1 in 5 patients with hip fracture, while broader cognitive impairment is even more common. In the acute setting, pre-existing cognitive impairment may be difficult to distinguish from delirium, complicating assessment of prognosis, decision-making capacity, and expected treatment benefit [7]. For patients who are permanently immobile or near the end of life, surgeons may select IF over guideline-recommended arthroplasty as a shorter, less invasive means of providing fracture stabilization and pain relief while reducing operative burden [8].

Randomized clinical trials remain the preferred design for estimating treatment effects but may not fully represent the heterogeneous patients and treatment settings encountered in routine hip fracture care. Target trial emulation can complement trial evidence by explicitly defining the eligibility criteria, treatment strategies, time zero, follow-up, outcomes, causal contrast, and analysis of the hypothetical trial if baseline data can be made comparable and similar follow-up takes place [9,10]. Using nationwide Danish registry data, we emulated a target trial of arthroplasty vs internal fixation (IF). The primary objective was to estimate the intention-to-treat effect of assignment to arthroplasty vs IF on the cumulative incidence of recorded reoperation within 365 days among patients aged ≥ 70 years with dementia and displaced FNF. Additional outcomes were major reoperation, all-cause mortality, length of hospital stay, early mobilization, and postoperative mobility loss.

## Methods

### Study design and setting

We conducted a Danish nationwide comparative-effectiveness study using routinely collected data from January 2012 to November 2021 to emulate a hypothetical pragmatic target trial. The target trial would compare assignment to arthroplasty vs IF as the initial surgical treatment of displaced FNF among patients aged ≥ 70 years with dementia. The primary estimand was the effect of assignment to arthroplasty vs IF on the cumulative incidence of recorded reoperations within 365 days among all eligible patients. Time zero was defined as the date of primary hip fracture surgery.

Denmark has a universal tax-funded healthcare system, and primary hip fracture surgery and subsequent reoperations are performed within public hospitals. We first specified the protocol of the hypothetical target trial, including eligibility criteria, treatment strategies, treatment assignment, time zero, follow-up, outcomes, causal contrast, and analysis (Supplementary Table S1). We then emulated each component using linked Danish healthcare registries [10,11].

The study is reported in accordance with the Strengthening the Reporting of Observational Studies in Epidemiology (STROBE), Reporting of Studies Conducted Using Observational Routinely Collected Health Data (RECORD), and Transparent Reporting of Observational Studies Emulating a Target Trial (TARGET) guidelines [9,12,13].

### Data sources

Patients were identified from the Danish Multidisciplinary Hip Fracture Registry (DMHFR), which has systematically collected data since 2004 on all patients aged ≥ 65 years undergoing primary hip fracture surgery. Reporting is mandatory for all Danish public hospitals. The positive predictive value for recorded hip fracture events is 100%, and registry audits indicate approximately 90% completeness for quality indicators and descriptive variables, including Cumulated Ambulation Score (CAS), body mass index (BMI), and mobilization [3].

All Danish residents are assigned a unique Civil Personal Register (CPR) number at birth or upon immigration, enabling individual-level, continuously updated linkage across nationwide health registries and virtually complete follow-up with accurate censoring at death or emigration [14].

The Danish National Patient Registry (DNPR), including the Danish Psychiatric Central Research Register (DPCRR), contains data on all hospital contacts, including admission and discharge dates, diagnosis codes, and surgical procedures [15]. The DNPR was used to identify comorbidities, mental disorders, treatments during hospitalization, and surgical procedures.

Data on the highest achieved educational level was obtained from Statistics Denmark through the National Population Education Register [16].

### Eligibility criteria

The source population comprised patients recorded in the DMHFR. We identified patients with displaced FNFs and excluded patients with non-displaced femoral neck, trochanteric, or subtrochanteric fractures. The cohort was restricted to patients aged ≥ 70 years. Patients without a dementia diagnosis, defined by ICD-10 codes F00–F03 or G30 and recorded in the DNPR or DPCRR within 10 years before surgery, were excluded (see Figure 1).

**Figure 1:**
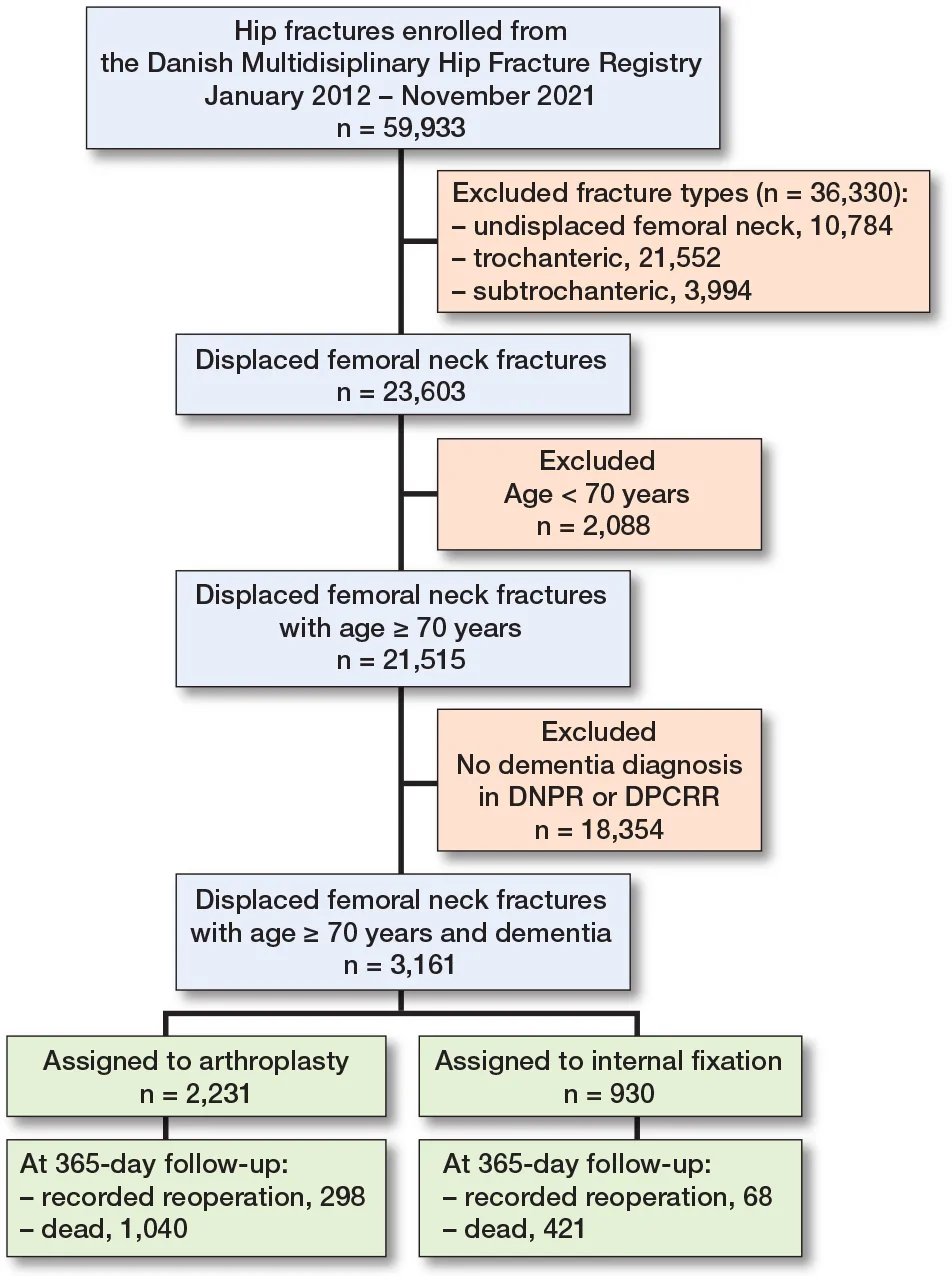
Flow of patients through the emulated target trial comparing arthroplasty with internal fixation in adults aged ≥ 70 years with dementia and displaced femoral neck fracture. DNPR = Danish National Patient Registry; DPCRR = Danish Psychiatric Central Research Register. Dementia was defined using ICD-10 codes F00–F03 or G30.

### Treatment assignment and strategies

The target trial compared 2 surgical treatments: arthroplasty and IF. Treatment was defined by the primary procedure received at time zero. Patients were analyzed according to this initial treatment strategy, consistent with an intention-to-treat estimand.

### Outcomes

The primary outcome was hip-related reoperation within 365 days. Procedure-specific definitions were used to identify reoperations following arthroplasty and IF. Surgical procedures were identified using NOMESCO Classification of Surgical Procedures (NCSP codes) recorded in the Danish National Patient Registry (Supplementary Table S2).

A reoperation was defined as any subsequent hip-related surgical intervention recorded in the Danish National Patient Registry, including closed reduction of prosthetic dislocation.

Secondary outcomes were major reoperation and all-cause mortality. Major reoperation was defined using a more restrictive outcome definition that excluded NCSP code NFH20, representing closed reduction of a dislocated prosthesis. NCSP code NFU49, representing removal of internal fixation material, was also excluded when performed more than 6 months after the index procedure and recorded together with ICD-10 code T84.1, because these procedures were considered more likely to reflect hardware-related symptoms than structural failure of the fixation.

### Follow-up

Follow-up began at time zero and continued until the 1st recorded reoperation, death, emigration, completion of 365 days of follow-up, or the end of available data, whichever occurred first. For analyses of reoperation, death was treated as a competing event rather than as an independent censoring event.

The intention-to-treat analysis classified patients according to their initial surgical treatment throughout follow-up. A separate per-protocol analysis was not specified because changing the initial surgical treatment would require a reoperation, which was the primary outcome and ended follow-up.

### Covariates

To characterize the study population at time zero, we obtained information on demographic factors, including sex and age; BMI; socioeconomic factors, including education level, marital status, and living situation; comorbidities, including cardiovascular, hepatic or gastrointestinal, malignant, musculoskeletal, neurological or alcohol-related, pulmonary, renal or hematological, and other mental disorders excluding dementia; and treatment-related variables, including calendar year of surgery, time to surgery, region of treatment, and hospital of treatment (see Table 1, Supplementary Table S3).

**Table 1:** Baseline characteristics of the unweighted target trial emulation cohort. Values are n (%) or mean (SD)

Factor	Arthroplasty (n = 2,231)	Internal fixation (n = 930)	Absolute standardized mean difference	
			crude	weighted
Female	1,630 (73)	641 (69)	0.04	0.01
Age, mean (SD)	85 (6.4)	85 (6.2)	0.07	0.00
BMI, mean (SD)	23 (4.2)	23 (4.4)	0.05	0.00
Surgery year			0.21	0.02
2012–2014	691 (31)	358 (39)	-	-
2015–2017	685 (31)	307 (33)	-	-
2018–2021	855 (38)	265 (29)	-	-
Hours to surgery, mean (SD)	23.0 (16.4)	20.9 (14.8)	-	-
Education level				
None	130 (5.8)	62 (6.7)	0.01	0.00
Low	1,137 (51)	510 (55)	0.04	0.01
Medium	697 (31)	277 (30)	0.01	0.01
High	267 (12)	81 (8.7)	0.03	0.00
Marital status				
Married	643 (29)	260 (28)	0.01	0.00
Divorced	289 (13)	116 (13)	0.00	0.00
Widow(er)	1,194 (54)	494 (53)	0.00	0.00
Never married	105 (4.7)	60 (6.5)	0.02	0.00
Living situation				
Home, cohab	303 (14)	117 (13)	0.01	0.00
Home, alone	378 (17)	152 (16)	0.01	0.01
Nursing home	1,418 (64)	594 (64)	0.00	0.01
Other	132 (5.9)	67 (7.2)	0.01	0.00
Comorbidity				
Cardiovascular	1,347 (60)	585 (63)	0.03	0.00
Hepatic/gastro-intestinal	103 (4.6)	46 (4.9)	0.00	0.00
Malignant	335 (15)	142 (15)	0.00	0.00
Mental disorders	255 (11)	109 (12)	0.00	0.00
Metabolic	545 (24)	248 (27)	0.02	0.00
Musculoskeletal	99 (4.4)	35 (3.8)	0.01	0.00
Neurological/alcohol	276 (12)	116 (13)	0.00	0.00
Pulmonary	258 (12)	144 (16)	0.04	0.00
Renal/hematological	706 (32)	321 (35)	0.03	0.00
Region				
Capital	699 (31)	226 (24)	0.07	0.00
Central	426 (19)	147 (16)	0.03	0.00
Northern	230 (10)	117 (13)	0.02	0.00
Southern	581 (26)	303 (33)	0.07	0.00
Zealand	295 (13)	137 (15)	0.02	0.00

The table presents numbers before imputation.

The percentage of missing values before imputation was as follows: body mass index, 18%.

Treatment with arthroplasty and internal fixation was weighted according to sex, age, body mass index, surgery year, education level, marital status, living situation, comorbidities (cardiovascular, hepatic/gastrointestinal, malignant, musculoskeletal, neurological/alcohol pulmonary, renal/hematological, mental disorders [without dementia]), region of treatment, and hospital of treatment.

Comorbidities were identified using primary and secondary hospital discharge diagnoses recorded during inpatient and outpatient contacts within a 10-year lookback period before the index surgery, to capture both recent and long-standing chronic conditions.

### Statistics

The primary analysis estimated the intention-to-treat average treatment effect of assignment to arthroplasty vs IF on the cumulative incidence of recorded reoperations within 365 days.

Population characteristics at time zero were described separately for the 2 treatment strategies using means with 95% confidence intervals (CIs) for continuous variables and counts with percentages for categorical variables. Missing BMI values for 585 of 3,161 patients (18%) were handled using multiple imputation by fully conditional specification, generating 10 imputed datasets [17]. The imputation model included all variables used in the treatment model.

To approximate baseline exchangeability between the treatment strategies, stabilized inverse probability of treatment weights (IPTW) was estimated separately within each imputed dataset. Logistic regression was used to estimate each patient’s probability of receiving the observed treatment conditional on the prespecified baseline covariates. The weights targeted the average treatment effect in the eligible study population [18].

The covariates included in the treatment model are presented in Table 1, and the distribution of weights, including the minimum, 1st quartile, median, mean, 3rd quartile, and maximum, in Supplementary Table S4.

Covariate balance after weighting was assessed using absolute standardized mean differences, with values below 0.1 considered indicative of acceptable balance [19].

Crude and weighted cumulative incidences of recorded reoperation were estimated using the Aalen–Johansen estimator, with death treated as a competing event. Curves were plotted over 365 days, and absolute risks and risk differences were estimated at 30 and 365 days. The cumulative incidence of all-cause mortality was estimated using the Kaplan–Meier estimator. Crude and weighted cause-specific Cox proportional hazards models were fitted to estimate hazard ratios (HRs), with arthroplasty as the reference treatment.

Non-parametric bootstrapping with 500 resamples was used to obtain percentile-based CIs for the effect estimates [20]. Bootstrapping was performed within each imputed dataset using case resampling, with the weighted outcome estimates recalculated in each bootstrap sample. Estimates were averaged across imputations within each bootstrap replicate, and percentile limits were derived from the resulting bootstrap distribution. Point estimates were combined across the imputed datasets using Rubin’s rules [20].

Additional outcomes were postoperative length of stay, mobilization within 24 h, and mobility loss, defined as a reduction in CAS from pre-fracture to postoperative assessment. Mobility analyses were restricted to patients with recorded CAS. IPTW was used to estimate treatment-specific means or proportions and the corresponding differences. Continuous outcomes were analyzed using weighted linear regression and binary outcomes using weighted generalized linear models with an identity link. Percentile-based CIs were obtained using 500 non-parametric bootstrap resamples. In additional analyses, we described the distribution of arthroplasty procedures according to cementation status and extended follow-up for reoperation to 2 years to capture later complications after internal fixation.

All analyses were conducted using R, version 4.3.2 (R Foundation for Statistical Computing, Vienna, Austria).

### Ethics, data sharing plan, use of AI, funding, and disclosures

The study was reported to the Danish Data Protection Agency through Aarhus University (Record number AU-2016-051-000001, sequential number 880). The data analyzed in this study was made available in anonymized form on Statistics Denmark’s servers. To protect the privacy of patients, individual-level data is not publicly disclosed, but access may be granted upon application to the relevant Danish authorities. AI-based tools were used solely to assist with grammatical and punctuation improvements. SSJ received funding from the Health Research Fund of the Central Denmark Region (grant paid to Aarhus University). The funding source was not involved in the study design, data analysis, interpretation of results, preparation, or submission of the manuscript. The authors declare no conflicts of interest. Complete disclosure of interest forms according to ICMJE are available on the article page, doi: 10.2340/17453674.2026.46936

## Results

### Study population and baseline characteristics

The emulated target trial included 3,161 patients, of whom 2,231 received arthroplasty, and 930 received internal fixation (Figure 1). Compared with patients treated with arthroplasty, those treated with internal fixation had a shorter mean time to surgery, were more often male, and had a slightly lower educational level. Other measured characteristics were broadly comparable. After IPTW, all measured covariates were balanced between the treatment groups, with absolute standardized mean differences below 0.02 (Table 1 and Supplementary Figure S1).

### Primary outcome

Within 30 days, recorded reoperations occurred in 201 of 2,231 patients after arthroplasty and 22 of 930 after internal fixation. The IPTW-adjusted cumulative incidences were 9.1% (CI 8.8–9.5) and 2.4% (CI 2.1–2.7), respectively, corresponding to an absolute difference of −6.7 percentage points (CI −7.2 to −6.2) and an HR of 0.24 (CI 0.15–0.39). Within 365 days, recorded reoperations occurred in 298 of 2,231 patients after arthroplasty and 68 of 930 after internal fixation. The IPTW-adjusted cumulative incidences were 13% (CI 13–14) and 7.6% (CI 7.0–8.3), respectively, corresponding to an absolute difference of −5.8 percentage points (CI −6.5 to −5.0) and an HR of 0.52 (CI 0.39–0.69). The crude and IPTW-adjusted estimates were similar at both time points (Figure 2 and Table 2).

**Figure 2:**
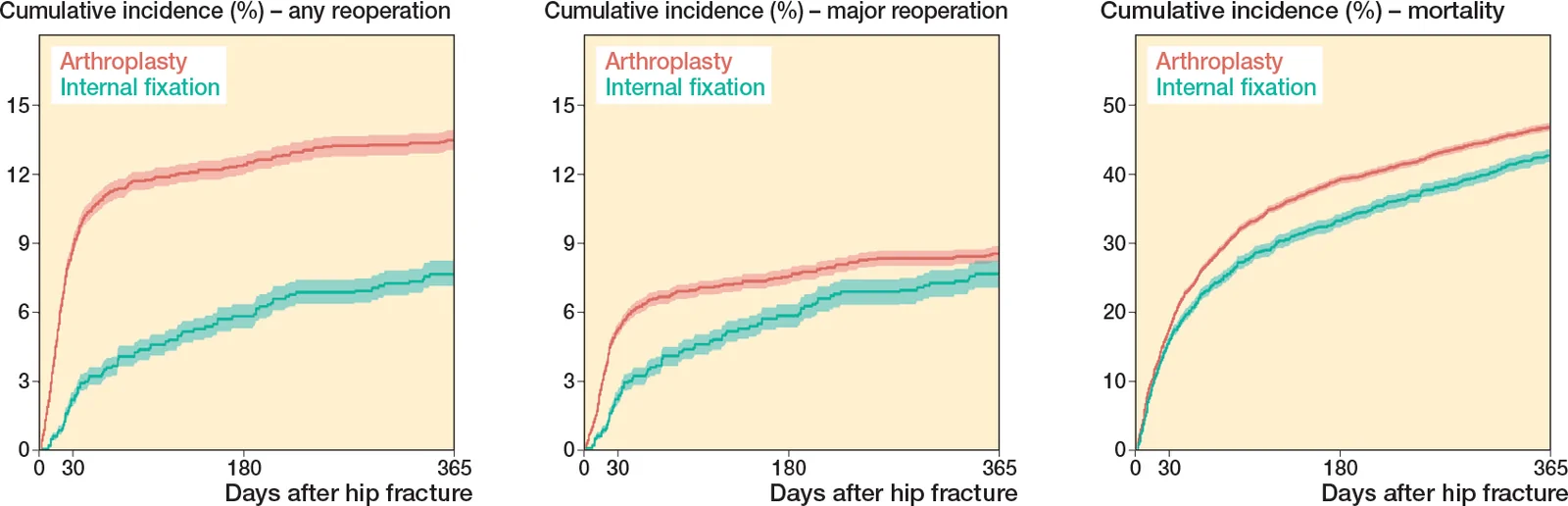
Cumulative incidence of recorded reoperation, major reoperation, and all-cause mortality after arthroplasty vs internal fixation among patients aged ≥ 70 years with dementia and displaced femoral neck fracture. The treatment groups were weighted according to sex, age, body mass index, surgery year, education level, marital status, living situation, comorbidities (cardiovascular, hepatic/gastrointestinal, malignant, musculoskeletal, neurological/alcohol pulmonary, renal/hematological, mental disorders [without dementia]), region of treatment, and hospital of treatment.

**Table 2:** Crude and IPTW-adjusted estimates of reoperation and mortality after arthroplasty vs internal fixation

Analyses	Follow-up days	Arthroplasty (n = 2,231) % (CI)	Internal fixation (n = 930) % (CI)	Absolute difference, percentage points (CI)	HR (CI)
Crude					
Any reoperation ^**a**^	30	9.0 (7.8–10)	2.4 (1.4–3.3)	−6.6 (−8.2 to −5.1)	0.25 (0.16–0.39)
Any reoperation ^**a**^	365	13 (12–15)	7.3 (5.6–9.0)	−6.0 (−8.2 to −3.9)	0.51 (0.39–0.67)
Major reoperation ^**a**^	30	5.2 (4.3–6.2)	2.4 (1.4–3.3)	−2.9 (−4.2 to −1.5)	0.44 (0.28–0.70)
Major reoperation ^**a**^	365	8.6 (7.4–9.7)	7.3 (5.6–9.0)	−1.2 (−3.3 to 0.8)	0.83 (0.63–1.09)
All-cause mortality	30	18 (16–20)	18 (15–20)	−0.2 (−3.1 to 2.8)	0.99 (0.83–1.18)
All-cause mortality	365	47 (45–49)	45 (42–49)	−1.3 (−5.2 to 2.5)	0.95 (0.85–1.06)
IPTW-adjusted					
Any reoperation ^**a**^	30	9.1 (8.8–9.5)	2.4 (2.1–2.7)	−6.7 (−7.2 to −6.2)	0.24 (0.15–0.39)
Any reoperation ^**a**^	365	13 (13–14)	7.6 (7.0–8.3)	−5.8 (−6.5 to −5.0)	0.52 (0.39–0.69)
Major reoperation ^**a**^	30	5.3 (5.0–5.6)	2.4 (2.1–2.7)	−3.0 (−3.3 to −2.5)	0.43 (0.27–0.70)
Major reoperation ^**a**^	365	8.6 (8.2–8.9)	7.6 (7.1–8.2)	−1.0 (−1.7 to −0.2)	0.84 (0.63–1.13)
All-cause mortality	30	18 (18–19)	16 (16–17)	−2.0 (−3.0 to −1.1)	0.88 (0.73–1.06)
All-cause mortality	365	47 (46–48)	43 (42–44)	−4.3 (−5.5 to −3.0)	0.86 (0.77–0.98)

The treatment groups were weighted, see Table 1

CI = 95% confidence interval; HR = hazard ratio; IPTW = inverse probability of treatment weighting.

a Cumulative incidence for reoperation, treating death as a competing risk.

### Secondary outcomes

#### Major reoperations

Within 30 days, major reoperations occurred in 117 patients after arthroplasty and 22 after internal fixation. The IPTW-adjusted cumulative incidences were 5.3% (CI 5.0–5.6) and 2.4% (CI 2.1–2.7), respectively. Within 365 days, major reoperations occurred in 191 and 68 patients, with corresponding IPTW-adjusted cumulative incidences of 8.6% (CI 8.2–8.9) and 7.6% (CI 7.1–8.2). The adjusted HRs were 0.43 (CI 0.27–0.70) at 30 days and 0.84 (CI 0.63–1.10) at 365 days (see Table 2).

#### Mortality

Within 30 days, 402 patients treated with arthroplasty and 166 patients treated with IF had died. The crude mortality was 18% in both groups, whereas the IPTW-adjusted estimates were 18% (CI 18–19) and 16% (CI 16–17), respectively. Within 365 days, 1,040 and 421 patients had died after arthroplasty and IF, respectively. The crude mortality estimates were 47% (CI 45–49) and 45% (CI 42–49), whereas the IPTW-adjusted estimates were 47% (CI 46–48) and 43% (CI 42–44), respectively. The adjusted HRs were 0.88 (CI 0.73–1.10) at 30 days and 0.86 (CI 0.77–0.98) at 365 days.

### Additional outcomes and analyses

Patients treated with arthroplasty had a modestly longer hospital stay than those treated with internal fixation, with weighted means of 6.0 days (CI 5.7–6.3) and 5.2 days (CI 4.9–5.5), respectively. Early mobilization, postoperative mobility loss, and mean reduction in CAS were similar between the treatment strategies (Table 3). Among patients treated with arthroplasty, 56% received an uncemented stem (Supplementary Table S5). Extending follow-up to 2 years showed that any reoperation remained more frequent after arthroplasty than after IF, whereas major reoperation remained similar between groups. Mortality increased substantially in both groups, while the difference between them narrowed (Supplementary Figure S2).

**Table 3:** Observed and IPTW-adjusted estimates of additional outcomes after arthroplasty vs internal fixation. Observed values are presented as n/N (%) for binary outcomes and N for continuous outcomes. Denominators for binary outcomes include patients with available outcome data

Outcome	Arthroplasty, observed n/N (%) or N	Arthroplasty, IPTW-adjusted estimate (CI)	Internal fixation, observed n/N (%) or N	Internal fixation, IPTW-adjusted estimate (CI)	Adjusted difference (CI)
Length of hospital stay	2,231	6.0 days (5.7–6.3)	930	5.2 days (4.9–5.5)	–0.8 days (–1.2 to –0.4)
Early mobilization initiated	1,766/2,074 (85)	78.4% (76.7–80.3)	743/875 (85)	81.4% (78.9–83.9)	3.0% (0 to 6)
Mobility loss by CAS score	1,445/1,780 (81)	81.0% (79.2–82.7)	579/709 (82)	81.6% (78.6–84.3)	1.1% (–3 to 4)
Mean mobility loss by CAS	1,780	2.5 points (2.4–2.6)	709	2.5 points (2.3–2.6)	0.05 points (–0.19 to 0.10)

The treatment groups were, see Table 1.

CAS = Cumulated Ambulation Score; CI = 95% confidence interval; IPTW = inverse probability of treatment weighting.

## Discussion

We emulated a target trial of arthroplasty vs internal fixation (IF) on the cumulative incidence of recorded reoperation within 365 days among patients aged ≥ 70 years with dementia and displaced FNF. We found that IF was associated with a lower 365-day cumulative incidence of recorded reoperation than arthroplasty, with the largest difference in the early postoperative period. The difference was attenuated for major reoperations, suggesting that acute arthroplasty-related complications may have contributed to the observed pattern. Mortality was slightly lower after IF, while functional outcomes were similar and length of stay was modestly shorter. Target trial emulation provided a feasible and transparent framework for comparing arthroplasty with IF using nationwide routinely collected data. The lower risks of both recorded reoperation and mortality after IF were unexpected, suggesting that treatment implementation, residual confounding, and differential ascertainment of complications may have influenced the findings.

Two Swedish randomized trials in patients with displaced FNF and severe cognitive dysfunction have reported reoperation rates of 23–33% after IF, compared with 3.3% after cemented well-documented hemiarthroplasty using a direct lateral approach [21,22]. Hip function, complications, and mortality were similar, although health-related quality of life was worse after hemiarthroplasty at final follow-up. By contrast, our 13% estimate of recorded reoperations after arthroplasty was similar to that reported after uncemented Austin Moore hemiarthroplasty [23], whereas the 7.6% estimate after IF was substantially lower than trial estimates reported in the Swedish studies. This discrepancy may reflect frequent use of posterior approaches and uncemented stems in arthroplasties performed in Denmark, together with incomplete ascertainment of gradually developing failures after IF.

A small, older Dutch study randomized patients with dementia to internal fixation or hemiarthroplasty and reported high mortality and poor functional recovery after both procedures [24]. The hemiarthroplasty group received a cemented Thompson monoblock prosthesis through an anterior approach. A retrospective German study of patients undergoing hemiarthroplasty with an uncemented Corail AMT stem and bipolar head reported poorer postoperative mobility and higher revision rates among patients with dementia than among those without dementia [23]. Both studies were small, and the treatment approaches used differed from contemporary guideline-recommended practice, limiting the direct applicability of their findings.

Two Norwegian registry studies have reported partly divergent findings. The older study reported fewer reoperations and better patient-reported outcomes after hemiarthroplasty than after internal fixation in older patients with displaced FNF, including a sub-analysis of patients with cognitive impairment, while mortality did not differ between treatment groups [25]. A more recent study found that, compared with cognitively intact patients, cognitively impaired patients had a markedly lower risk of reoperation after IF but a slightly higher risk after hemiarthroplasty, mainly due to dislocation and periprosthetic fracture [26]. The apparent divergence should be interpreted based on the different comparisons: the older study compared IF with hemiarthroplasty for displaced FNF, whereas the more recent study compared patients with and without cognitive impairment within treatment groups across hip fracture types.

The 1-year mortality of 43–47% was substantially higher than the approximately 24% reported in general Scandinavian hip fracture populations [27]. This was expected and consistent with previous literature, as the present cohort was restricted to older individuals with dementia [26,28].

### Explanation of findings

The frequent use of the posterior approach and uncemented stems may have contributed to the observed reoperation risk after arthroplasty. The continued use of uncemented stems may also reflect incomplete uptake of evidence favoring cemented fixation. These procedure-related factors should be distinguished from the comparative effectiveness of arthroplasty and IF.

The study also highlights the importance of follow-up when recorded reoperation is used as an outcome. Routine clinical and radiographic follow-up after hip fracture surgery is not nationally standardized in Denmark. Reliance on patients returning with symptoms may be particularly problematic among people with dementia, in whom gradually developing complications after IF, such as non-union and avascular necrosis, may remain undetected [26,28]. In contrast, dislocation and periprosthetic fracture after arthroplasty usually present acutely, while infection may also produce observable signs. These complications may therefore be recognized even when patients cannot report symptoms.

The high mortality and similar functional outcomes in both treatment groups also highlight that, for selected patients with advanced dementia or frailty, permanent immobility, or limited life expectancy, the relevant decision may extend beyond treatment strategy [29]. For patients with a very limited life expectancy, treatment decisions should prioritize pain relief while considering the operative burden, goals of care, and the wishes of the patient and their relatives [30]. In this exceptional group, cemented arthroplasty using a dislocation-preventive technique or internal fixation may be considered, while nonoperative palliative management is generally reserved for patients with an extremely short expected survival [6]. Primary excision arthroplasty has also been proposed in selected cases but remains controversial and is not standard practice [5,31].

The active-comparator target trial emulation provided a structured framework for defining the clinical question and aligning eligibility criteria, treatment strategies, time zero, follow-up, outcomes, and analysis in observational data. This enabled comparison of implemented routine care rather than under optimized trial conditions. Target trial emulation can complement randomized evidence by examining gaps between trial efficacy, treatment implementation, and recorded effectiveness in routine care. However, causal interpretation remains dependent on the quality and completeness of the underlying data, and equal follow-up, which may be the main limitation of the present study. Thus, the framework cannot compensate for clinically important outcomes that are not captured.

### Strengths

The active-comparator design addressed the clinical choice between arthroplasty and IF, while IPTW balanced measured baseline characteristics. However, target trial emulation cannot overcome incomplete measurement of treatment-selection factors, which may result in residual confounding, or differential ascertainment of treatment failure when recorded reoperation is used as the outcome [32].

### Limitations

The principal limitation concerns the use of recorded reoperation as a proxy for treatment failure in this population. Recorded reoperation is an objective and clinically important outcome, but its use as a proxy for treatment failure is a principal limitation in this population. Among patients with dementia, complications may remain unrecognized or may not lead to further surgery because of impaired symptom reporting, competing mortality, or a higher threshold for reoperation. As discussed earlier, differences in how complications present and are detected may have made recorded reoperation a more sensitive proxy for treatment failure after arthroplasty than after IF, resulting in differential outcome ascertainment between the treatment strategies [32].

The estimated reoperation difference should also be interpreted in the context of how the treatment strategies were implemented in routine Danish clinical practice during the study period. Arthroplasties were commonly performed using a posterior approach and uncemented stems, which may have contributed to increased risk of dislocations and periprosthetic fractures. The estimated treatment effect may therefore differ in settings where cemented stems, anterolateral or direct lateral approaches, or other dislocation-preventive measures are more commonly used [33].

Residual confounding remains possible despite adjustment using IPTW. Information on frailty, dementia severity, ASA classification, and aspects of functional status beyond the recorded measures were unavailable. Patients with milder dementia and longer expected survival may have been preferentially selected for arthroplasty, whereas IF may have been used as a less invasive strategy in patients with more advanced dementia, limited rehabilitation potential, or short expected survival. Such selection could have contributed to fewer recorded reoperations after IF through competing mortality or a higher threshold for further surgery. However, it would not readily explain the lower mortality observed after IF, suggesting that treatment selection was more complex and that the direction of residual confounding cannot be determined. Dementia was identified from hospital-based diagnoses in the DNPR. This approach may underrepresent milder cases managed exclusively in primary care and does not distinguish dementia severity among included patients.

Although we adjusted for treatment region and hospital to account for systematic regional and institutional variation, individual surgeon-level decision-making could not be measured directly. Patient-reported outcomes were unavailable, and we could not assess pain or quality of life [5]. This is particularly relevant among patients with dementia, in whom persistent pain, impaired mobility, or poor functional recovery may not necessarily lead to surgical revision [34,35]. Accordingly, the estimated effect should be interpreted as the effect of treatment assignment on recorded reoperation rather than on all underlying treatment failures.

### Conclusion

IF was associated with fewer recorded reoperations and lower mortality than arthroplasty, while functional outcomes were similar.

The findings illustrate that target trial emulation can strengthen design transparency but cannot overcome treatment-specific implementation, unmeasured treatment-selection factors, or differential ascertainment of complications. These limitations should be considered when interpreting estimates from target trial emulations using routinely collected clinical data.

### Supplementary data

Figures S1–S2 and Tables S1–S4 are available as supplementary data on the article page, doi: 10.2340/17453674.2026.46936

## Supplementary Material



## Notes

SSJ, PHG, RN, JEG, and ABP conceived and designed the study. SSJ and ABP contributed to the statistical analysis, and accessed and verified the data. SSJ and ABP wrote the paper. SSJ, PHG, RN, JEG, and ABP revised the paper critically for important intellectual content. SSJ, PHG, RN, JEG, and ABP were responsible for final approval and agreement of accountability for all aspects of the work.

Handling co-editors: Cecilia Rogmark and Robin Christensen

*Acta* thanks Frede Frihagen and Linnea Malmgren for help with peer review of this manuscript.
