# Supplementary material for: Arthroplasty versus internal fixation for displaced femoral neck fractures in older patients with dementia: a target trial emulation study
Source: Acta Orthop. 2026 Oct 26;97:758–66. doi: 10.2340/17453674.2026.46936 (PMC13697299; doi:10.2340/17453674.2026.46936)
Supplement: Supplementary file 1 [file ActaO-97-46936-s1.pdf]

Online Supplementary Material

Journal

Acta Orthopaedica

Article title

Arthroplasty versus internal fixation for displaced femoral neck fractures in older patients with dementia: a target trial emulation study

Authors

Simon Storgaard Jensen [1,2], Per Hviid Gundtoft [3], Rob G H H Nelissen [4], Jan-Erik Gjertsen [5,6], and Alma B Pedersen [1,2]

Affiliation

[1] Department of Clinical Epidemiology, Aarhus University Hospital, Aarhus, Denmark.
[2] Department of Clinical Medicine, Aarhus University, Aarhus, Denmark.
[3] Department of Orthopaedic Surgery, Traumatology, Aarhus University Hospital, Aarhus, Denmark.
[4] Department of Orthopaedics, Leiden Universitair Medisch Centrum, Leiden, The Netherlands
[5] Department of Orthopaedic Surgery, Haukeland University Hospital, Bergen, Norway.
[6] Department of Clinical Medicine, University of Bergen, Norway.

Correspondence: Simon Storgaard Jensen, ssj@clin.au.dk

**Supplementary Table 1. Target trial specification and emulation**			
**Protocol component**	**Target trial protocol**	**Target trial emulation**	**Data sources**
Aim	To estimate the effect of arthroplasty versus internal fixation on the risk of reoperation and mortality among adults aged ≥70 years with displaced femoral neck fractures and dementia.	Same as target trial	-
Eligibility Criteria	1) Adults aged ≥70 years. 2) First-time, unilateral hip fracture. 3) Displaced femoral neck fracture (S72.0 + ZDS01). 4) Dementia present at baseline (pre-fracture), ascertained by clinical assessment and/or medical records before the index operation.	Same as target trial, except:<br><br>4) Hospital diagnosis of organic, including symptomatic, mental disorders (dementia) based on ICD-10 codes (F00–F03, G30) in DNPR within 10 years preceding the first hip fracture surgery.	The Danish Multidisciplinary Hip Fracture Registry<br><br>The Danish National Patient Registry
Exclusion criteria	1) Age <70 years. 2) Secondary or bilateral hip fracture. 3) Pertrochanteric or subtrochanteric hip fracture (S72.1 or S72.2). 4) Absence of Dementia at baseline (pre-fracture), ascertained by clinical assessment and/or medical records before the index operation.	Same as target trial, except:<br><br>4) No hospital diagnosis of dementia (ICD-10 F00–F03, G30) recorded in DNPR within 10 years preceding index surgery.	The Danish Multidisciplinary Hip Fracture Registry<br><br>The Danish National Patient Registry
Enrolment	January 1st 2012	Same as for the target trial	-
Treatment strategy	1) Arthroplasty (NFBx): hemiarthroplasty or total hip arthroplasty as the primary surgical management of the index hip fracture.<br>2) Internal fixation (NFJx): internal fixation as the primary surgical management of the index hip fracture.	Same as for the target trial	The Danish Multidisciplinary Hip Fracture Registry
Treatment assignment	Random allocation to one of the two strategies at baseline (time of index operation).<br><br>Eligible persons and treating clinicians will be aware of the assigned treatment	Patients are assigned to the treatment that is compatible with the surgery code in the DMFHR. We assume randomization conditional on baseline covariates.<br><br>Eligible persons and treating clinicians will be aware of the assigned treatment	The Danish Multidisciplinary Hip Fracture Registry
Outcomes	Primary: Composite outcome of reoperation defined as any secondary hip-related surgical intervention, including closed reduction of prosthesis dislocation. Major reoperations, excluding NFH20 and NFU49 (if 6 months after the index	Same as for the target trial.<br><br>Reoperations were identified in the DNPR using NOMESCO surgical procedure codes, in accordance with the Danish Clinical Quality Program - National Clinical Registries, designed to benchmark	The Danish National Patient Registry<br><br>The Danish Civil Registration System<br><br>The Danish Multidisciplinary Hip Fracture Registry

	surgery and coded with T84.1)<br><br>Secondary: Death by all-cause mortality<br><br>Additional: Functional outcomes as part of post-operative hospital care, including length of hospital stay, early mobilization, mobility loss, and degree of mobility loss.	hospital performance and support quality improvement initiatives	
Follow-up	Follow-up starts at treatment initiation, time zero (index hip fracture operation).<br><br>Intention-to-treat: participants are followed from baseline until the first occurrence of the outcome of interest, death, end of the study period (November 2021), or loss to follow-up, whichever occurs first.<br><br>Per-protocol: Follow-up is not defined, as treatment changes after hip fracture surgery typically occur only through reoperation (outcome).	Same as the target trial.	The Danish Multidisciplinary Hip Fracture Registry<br><br>The Danish National Patient Registry<br><br>The Danish Civil Registration System
Causal estimand	Intention-to-treat: the effect of being assigned to the treatment strategy. We assume no loss to follow up.<br><br>Per-protocol effect: n/a	Observational analog of intention-to-treat effects.<br><br>Per-protocol effect: n/a	-
Statistical analyses	Cumulative incidence curves (risks) for each treatment strategy are estimated using the Aalen-Johansen estimator, considering competing risk from death. Risks are estimated for 30- and 365-day follow-up per outcome, used to calculate risk differences and ratios. If differences in baseline characteristics are observed despite randomization, models are adjusted for baseline confounders using stabilized inverse probability of treatment weights (IPTW). A non-parametric bootstrap method would be employed to obtain standard errors for calculating normal approximation 95% confidence intervals for risk differences and ratios.	Same as for the target trial. The intention-to-treat analysis is adjusted for baseline confounders using IPTW.	-

Supplementary Figure 1. Covariate balance between arthroplasty and internal fixation before and after weighting

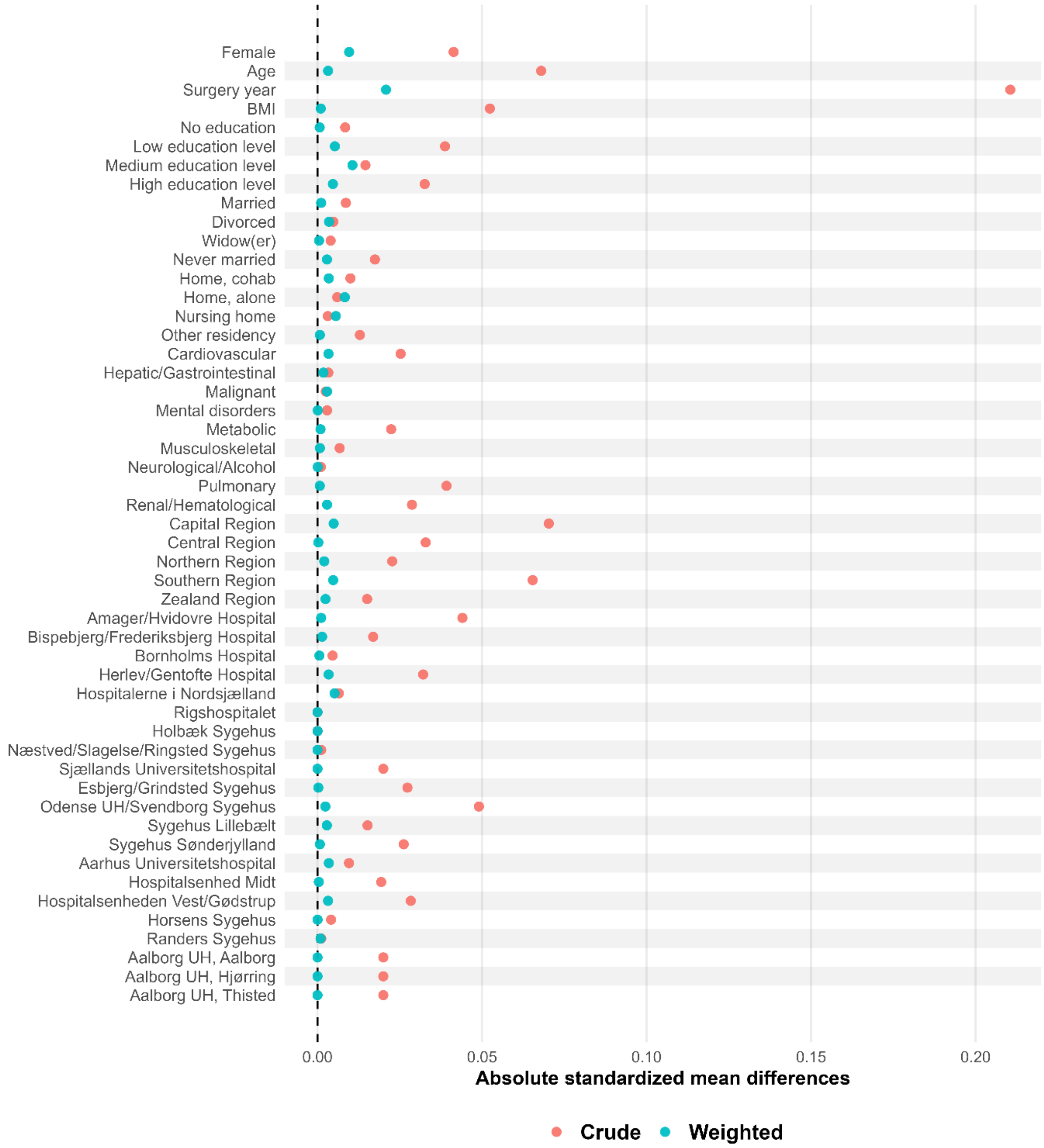

Note: The covariates used to calculate the weights included: Sex, age, body mass index (BMI), surgery year, education level, marital status, living situation, comorbidities (cardiovascular, hepatic/gastrointestinal, malignant, musculoskeletal, neurological/alcohol pulmonary, renal/hematological, mental disorders (without dementia)), region of treatment, and hospital of treatment.

Supplementary Table 2. – NOMESCO Classification of Surgical Procedures (NCSP) used for defining reoperations

Classification of reoperation from primary surgery	Examples and elaboration	NCSP Codes
Fracture of head and neck of femur, S72.0<br>+ Fracture surgery of femur, NFJ		
NFB0-99	Primary prosthetic replacement of hip joint	NFB, NFB0, NFB01, NFB02, NFB03, NFB09, NFB1, NFB11, NFB12, NFB13, NFB19, NFB20, NFB30, NFB40, NFB59, NFB99
NFC0-99	Secondary prosthetic replacement of hip joint	NFC, NFC0, NFC01, NFC02, NFC03, NFC09, NFC1, NFC11, NFC12, NFC13, NFC19, NFC2, NFC20, NFC21, NFC22, NFC23, NFC29, NFC3, NFC30, NFC31, NFC32, NFC33, NFC39, NFC4, NFC40, NFC41, NFC42, NFC43, NFC49, NFC59, NFC99
NFW0-99	Reoperations on hip or thigh	NFW, NFW49, NFW59, NFW69, NFW79, NFW89, NFW99
NFG09	Excision arthroplasty of hip joint	NFG09
NFU0-99	Removal of implants and external fixation devices from hip and femur	NFU, NFU0, NFU01, NFU02, NFU09, NFU1, NFU10, NFU11, NFU12, NFU19, NFU39, NFU49, NFU89, NFU89A, NFU89B, NFU99
NFS0-99	Operations for infection of tendons, joints and bone of hip and thigh	NFS, NFS19, NFS29, NFS49, NFS59, NFS99
Fracture of head and neck of femur, S72.0<br>+ Primary prosthetic replacement of hip joint, NFB		
NFB0-99	Primary prosthetic replacement of hip joint	NFB, NFB0, NFB01, NFB02, NFB03, NFB09, NFB1, NFB11, NFB12, NFB13, NFB19, NFB20, NFB30, NFB40, NFB59, NFB99
NFC0-99	Secondary prosthetic replacement of hip joint	NFC, NFC0, NFC01, NFC02, NFC03, NFC09, NFC1, NFC11, NFC12, NFC13, NFC19, NFC2, NFC20, NFC21, NFC22, NFC23, NFC29, NFC3, NFC30, NFC31, NFC32, NFC33, NFC39, NFC4, NFC40, NFC41, NFC42, NFC43, NFC49, NFC59, NFC99
NFW0-99	Reoperations on hip or thigh	NFW, NFW49, NFW59, NFW69, NFW79, NFW89, NFW99
NFG09	Excision arthroplasty of hip joint	NFG09
NFH0-29	Miscellaneous operations on hip joint	NFH, NFH0, NFH00, NFH01, NFH02, NFH2, NFH20, NFH21, NFH22, NFH29
NFU0-99	Removal of implants and external fixation devices from hip and femur	NFU, NFU0, NFU01, NFU02, NFU09, NFU1, NFU10, NFU11, NFU12, NFU19, NFU39, NFU49, NFU89, NFU89A, NFU89B, NFU99
NFS0-99	Operations for infection of tendons, joints and bone of hip and thigh	NFS, NFS19, NFS29, NFS49, NFS59, NFS99
Infection (T84.5 or T84.6 or T84.7) +		

S72.0 + NFB or NJF		
NFW69	Reoperation for deep infection in surgery of hip or thigh. For infection at the site of target structures	NFW69
NFS0-99	Operations for infection of tendons, joints and bone of hip and thigh	NFS, NFS19, NFS29, NFS49, NFS59, NFS99
NFU0-99	Removal of implants and external fixation devices from hip and femur	NFU, NFU0, NFU01, NFU02, NFU09, NFU1, NFU10, NFU11, NFU12, NFU19, NFU39, NFU49, NFU89, NFU89A, NFU89B, NFU99

Supplementary Table 3. – International Classification of Disease 10th Edition (ICD-10) codes used for defining comorbidities

**Comorbidities/Category**	**Examples and elaboration on comorbidities**	**ICD-10 Codes**
**Cardiovascular**		
Cerebrovascular disease	Ischemia and hemorrhagic stroke; Transient ischemic attack; Etc.	I60-I69; G45-G46
Heart arrhythmia	Atrioventricular block; Paroxysmal tachycardia; Atrial fibrillation and flutter; Etc.	I44.1 - I44.3; I45.6; I45.9; I47-I49; R00.0; R00.1; R00.8; T82.1; Z45.0; Z95.0
Heart failure	Hypertensive heart disease; Ischemic cardiomyopathy; Myocarditis; Chronic ischemic heart disease; Heart failure; Etc.	I11.0; I13.0; I13.2; I09.9; I25.5; I42.0; I42.5 - I43; I50; P29.0
Hypertension	Essential hypertension; Hypertensive heart disease or kidney disease; Secondary hypertension; Etc.	I10-I13; I15
Hypotension	Orthostatic hypotension; Etc.	I95; G90.9A
Myocardial infarction	Acute myocardial infarction; Complications to acute myocardial infarction; Etc.	I21-I23
Peripheral vascular disease	Atherosclerosis; Aortic or peripheral aneurysm and dissection; Arterial embolism and thrombosis; Claudication; Etc.	I70-I74; I77
Valvular heart disease	Mitral and aortic valve disease; etc.	A52.0; I05-I08; I09.1; I09.8; I34-I39; Q23.0-Q23.3; Z95.2-Z95.4
**Organic mental disorders (dementia)**		
Organic, including symptomatic, mental disorders (Dementia)	Includes dementia in Alzheimer's disease, vascular dementia, etc.	F00-F03, G30
**Hepatic/gastrointestinal**		
Liver disease	Hepatitis; Esophegael varices; Alcoholic liver disease; Toxic liver diesase; Hepatic failure; Liver cirrhosis; Etc.	B15.0; B16.0; B16.2; B18; B19.0; I85; K70.0-K70.4; K70.9; K71-K74; K76.0; K76.6
Peptic ulcer	Ulcer of esophagus; Gastric, duodenal or gastrojejunal ulcer; Etc.	K22.1; K25-K28
**Malignancy**		
Any solid tumor	Malignant neoplasms including brain cancer, lung cancer, breast cancer, melanoma; Etc.	C00-C75
Hematologic cancer	Lymphoma; Malignant myeloma; Leukemia; Etc.	C81-C85; C88; C90-C96
Metastatic solid tumor	Malignant neoplasm with metastasis; Etc.	C76-C80
**Metabolic**		
Diabetes, complicated	Type I and II diabetes with kidney, ophthalmic, circulatory complication; Etc.	E10.2-E10.8; E11.2-E11.8
Diabetes, uncomplicated	Type I and II diabetes without complications	E10.0, E10.1; E10.9, E11.0; E11.1; E11.9
Hypercholesterolemia	Hypercholesterolemia; Etc.	E78.0
T hyroid disease	Iodine-deficiency; Non-toxic goiter; Thyrotoxicosis; Hypothyroidism; Etc.	E00-05; E06.2; E89.0
**Mental disorders**		

Mental and behavioural disorders due to psychoactive substance use	Includes use of alcohol, cannabis, cocaine, nicotine, opioids, sedatives, hypnotics, anxiolytics, etc.	F10-F13; F19
Schizophrenia, schizotypal and delusional disorders	Includes schizophrenia, schizotypal disorders, schizoaffective disorders and other psychotic disorders.	F20-F29
Mood [affective] disorders	Manic disorders, bipolar disorders and major, moderate or mild Depression	F30; F31; F32.2; F32.3; F33.2; F33.3; F32.0; F32.1; F32.8; F32.9; F33.0; F33.1; F33.8; F33.9; F34; F38; F39
Neurotic, stress-related and somatoform disorders	Includes anxiety disorders, phobias, obsessive-compulsive disorders, etc.	F40-45; F48
Behavioral syndromes associated with physiological disturbances a nd physical factors	Includes anorexia nervosa, bulimia nervosa, etc.	F50.0; F50.2; F50.5; F50.9
Disorders of adult personality and behavior	Includes paranoid, schizoid, dissocial, emotionally unstable personality disorder, etc.	F60-F61
Mental retardation	Includes mild, moderate, severe mental retardation, etc.	F70-F79
Disorders of psychological development	Includes autism spectrum disorders	F82; F84
Behavioral and emotional disorders with onset usually occurring in childhood and adolescence	Includes attention-deficit hyperactivity disorder, conduct disorders, childhood emotional disorders, etc.	F90; F98.8; F98.9
**Musculoskeletal**		
Rheumatic disease	Sarcoidosis; Rheumatoid arthritis; Vasculitis; Systemic lupus erythematosus; Systemic sclerosis; Ankylosing spondylitis; Etc.	D86; L94.0; L94.1; L94.3; M05-M06; M08-M09; M12.0; M12.3; M30-M36; M45; M46.1; M46.8; M46.9
**Neurological/alcohol**		
Alcohol overuse	Mental and behavioral disorders due to use of alcohol; Niacin deficiency; Alcoholic cardiomyopathy; Alcoholic liver disease; Etc.	F10; E52; G62.1; I42.6; K29.2; K70.0; K70.3; K70.9; T51; Z50.2; Z71.4; Z72.1
Neurological disorders	Huntington's disease; Parkinson's disease; Multiple sclerosis; Epilepsy; Encephalopathy; Etc.	G10-G13; G20-G22; G25.4; G25.5; G31.2; G31.8; G31.9; G32; G35-G37; G40-G41; G93.1; G93.4; R47.0; R56
**Pulmonary**		
Chronic pulmonary disease	Bronchitis; Emphysema; Chronic obstructive pulmonary disease; Asthma; Pneumoconiosis; Interstitial lung disease; Etc.	J40-J47; J60-J67; J68.4; J70.1; J70.3; J84.1; J92.0; J96.1; J98.2; J98.3
Pulmonary circulation disorders	Pulmonary embolism; Pulmonary hypertension; Etc.	I26; I27; I28.0; I28.8; I28.9
**Renal/hematological**		
Anemia	Deficiency anemia; Blood loss anemia; Aplastic anemia; Etc.	D50-59; D60-64
Fluid and electrolyte disorders	Volume depletion; Disorders of fluid, electrolyte and acid-base balance; Etc.	E22.2; E86-E87
Renal disease	Hypertensive chronic kidney disease; Glomerular disease; Renal tubulo-interstitial diseases; Kidney failure; Etc.	I12-I13; N00-N05; N07; N11; N14; N17-N19; Q61

Supplementary Table 4. Median, mean, first quartile, third quartile, minimum, and maximum values of the inverse probability of treatment weights [a]

	Inverse probability of treatment weight					
	Mean	*Median*	*First quartile*	*Third quartile*	*Minimum*	*Maximum*
**Overall**	2.00	1.51	1.30	2.31	1.08	10.68
**Subgroup**						
Females	2.02	1.48	1.29	2.32	1.08	10.68
Males	1.95	1.59	1.35	2.29	1.12	8.53
No education	2.00	1.60	1.34	2.23	1.13	6.71
Low education level	2.02	1.56	1.34	2.34	1.12	8.38
Medium education level	1.96	1.47	1.29	2.30	1.09	8.53
High education level	2.05	1.35	1.22	1.63	1.08	10.68
Married	1.93	1.74	1.41	2.20	1.14	4.88
Divorced	1.99	1.49	1.29	2.29	1.10	8.12
Widow(er)	2.04	1.50	1.29	2.27	1.09	10.68
Never married	2.00	1.50	1.31	2.35	1.08	9.20
Home, cohab	2.04	1.46	1.29	2.34	1.11	7.66
Home, alone	1.93	1.46	1.28	2.28	1.09	9.20
Nursing home	2.01	1.51	1.31	2.32	1.08	8.53
Other residency	1.99	1.67	1.36	2.30	1.12	10.68
Cardiovascular (÷)	2.02	1.47	1.29	2.33	1.08	8.17
Cardiovascular (+)	1.99	1.53	1.32	2.30	1.09	10.68
Hepatic/Gastrointestinal (÷)	1.99	1.50	1.30	2.30	1.08	10.68
Hepatic/Gastrointestinal (+)	2.06	1.57	1.33	2.39	1.14	8.53
Malignant (÷)	2.01	1.50	1.31	2.31	1.08	10.68
Malignant (+)	1.99	1.53	1.29	2.30	1.10	7.94
Mental disorders (÷)	2.00	1.50	1.30	2.30	1.08	10.68
Mental disorders (+)	2.01	1.52	1.32	2.41	1.10	7.94
Metabolic (÷)	2.00	1.49	1.30	2.30	1.08	10.68
Metabolic (+)	2.00	1.55	1.34	2.32	1.10	8.53
Musculoskeletal (÷)	2.00	1.51	1.31	2.31	1.09	10.68
Musculoskeletal (+)	1.99	1.43	1.27	2.27	1.08	8.38
Neurological/Alcohol (÷)	2.00	1.51	1.30	2.32	1.08	10.68
Neurological/Alcohol (+)	2.01	1.50	1.30	2.23	1.09	7.58
Pulmonary (÷)	2.00	1.48	1.29	2.32	1.08	10.68
Pulmonary (+)	2.00	1.74	1.43	2.28	1.16	6.64
Renal/Hematological (÷)	2.01	1.48	1.29	2.31	1.09	10.68
Renal/Hematological (+)	1.99	1.56	1.34	2.30	1.08	8.38
Capital	2.03	1.35	1.23	2.00	1.09	10.68
Central	2.00	1.39	1.24	2.11	1.08	8.38
Northern	2.04	1.62	1.45	2.56	1.22	5.23
Southern	1.97	1.67	1.41	2.28	1.13	7.47
Zealand	1.98	1.53	1.41	2.54	1.17	5.81
Amager/Hvidovre Hospital	2.02	1.24	1.17	1.34	1.09	10.68
Bispebjerg/Frederiksbjerg Hospital	2.06	1.72	1.47	2.43	1.18	5.33
Bornholms Hospital	2.06	1.29	1.23	1.34	1.15	7.52
Herlev/Gentofte Hospital	1.95	1.25	1.19	1.38	1.10	8.11
Hospitalerne i Nordsjælland	2.10	1.44	1.33	2.76	1.17	5.97
Rigshospitalet	-	-	-	-	-	-
Holbæk Sygehus	-	-	-	-	-	-
Næstved/Slagelse/Ringsted Sygehus	1.96	1.37	1.32	2.90	1.12	5.31
Sjællands Universitetshospital	1.98	1.53	1.41	2.54	1.17	5.81
Esbjerg/Grindsted Sygehus	1.97	1.88	1.70	2.15	1.40	3.81
Odense UH/Svendborg Sygehus	1.97	1.78	1.54	2.31	1.29	3.55
Sygehus Lillebælt	1.94	1.56	1.43	2.44	1.26	4.13
Sygehus Sønderjylland	2.02	1.28	1.21	1.40	1.13	7.47
Aarhus Universitetshospital	1.89	1.55	1.39	2.42	1.25	3.71
Hospitalsenhed Midt	1.99	1.25	1.20	1.31	1.10	8.17
Hospitalsenheden Vest/Gødstrup	2.07	1.24	1.20	1.34	1.08	8.38
Horsens Sygehus	2.02	1.99	1.80	2.23	1.63	2.42
Randers Sygehus	2.04	1.50	1.40	2.78	1.16	4.92
Aalborg UH, Aalborg	2.00	1.29	1.22	1.45	1.13	8.52
Aalborg UH, Hjørring	2.08	1.90	1.63	2.48	1.28	4.00
Aalborg UH, Thisted	2.06	2.07	1.79	2.20	1.57	3.14

[a] The covariates used to calculate the weights included: Sex, age, body mass index, surgery year, education level, marital status, living situation, comorbidities (cardiovascular, hepatic/gastrointestinal, malignant, musculoskeletal, neurological/alcohol, pulmonary, renal/hematological, mental disorders (without dementia)), region of treatment, hospital of treatment.

Supplementary Figure 2. IPTW-weighted cumulative incidence of recorded reoperation, major reoperation, and all-cause mortality after arthroplasty versus internal fixation among patients aged ≥70 years with dementia and displaced femoral neck fracture.

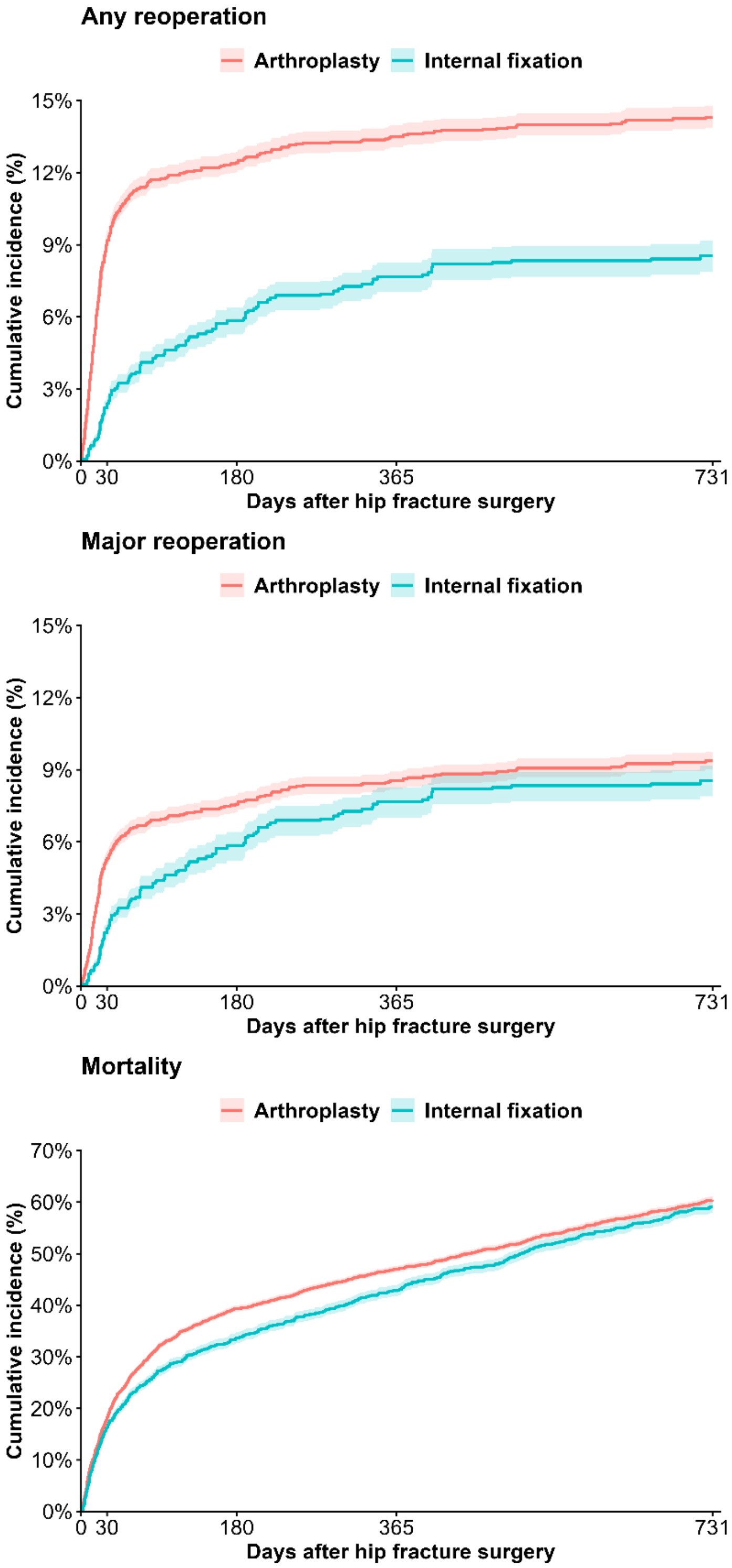

The treatment groups were weighted according to sex, age, BMI, surgery year, education level, marital status, living situation, comorbidities (cardiovascular, hepatic/gastrointestinal, malignant, musculoskeletal, neurological/alcohol pulmonary, renal/hematological, mental disorders (without dementia)), region of treatment, and hospital of treatment.

Supplementary Table 5. Distribution of arthroplasty procedures according to prosthesis type and stem fixation among patients with dementia and displaced femoral neck fracture

Arthroplasty procedure	n/N	%
NFB0,<br>Hemiarthroplasty, uncemented	1,196/2,231	54
NFB1,<br>Hemiarthroplasty, cemented.	743/2,231	33
NFB2,<br>Total hip arthroplasty, uncemented	38/2,231	1.7
NFB3,<br>Total hip arthroplasty, hybrid	188/2,231	8.4
NFB4,<br>Total hip arthroplasty, cemented	66/2,231	3.0

Values are presented as number (%). Percentages were calculated among patients treated with arthroplasty.
THA, total hip arthroplasty.
